# Tandem mass tag-based proteomic analysis reveals cathepsin-mediated anti-autophagic and pro-apoptotic effects under proliferative diabetic retinopathy

**DOI:** 10.18632/aging.202217

**Published:** 2020-12-03

**Authors:** Rui Niu, Jindan Wang, Chao Geng, Yahong Li, Lijie Dong, Lin Liu, Yuwen Chang, Jianqun Shen, Zetong Nie, Yan Zhang, Bojie Hu

**Affiliations:** 1 Tianjin Key Laboratory of Retinal Functions and Diseases, Tianjin International Joint Research and Development Centre of Ophthalmology and Vision Science, Eye Institute and School of Optometry, Tianjin Medical University Eye Hospital, Tianjin, China; 2 Hetian District People's Hospital, Xinjiang, China

**Keywords:** proliferative diabetic retinopathy, proteomics, cathepsin, CTSB, CTSD

## Abstract

Proliferative diabetic retinopathy (PDR) is a severe complication of diabetes and can cause blindness. However, the available therapeutic modalities to PDR have unsatisfactory efficacies and incur adverse effects, which is due to the paucity in the understanding of pathogenic mechanisms responsible for the disease. In this study, tandem mass tag labeling technology combined with liquid chromatography and tandem mass spectrometry were utilized to identify differentially expressed proteins in vitreous humor of patients with rhegmatogenous retinal detachment and PDR. The data are available via ProteomeXchange with identifier PXD021788. Afterwards, the downregulated protein expression of Cathepsin B, D, and L was verified in vitreous and serum of another cohort. The gene expression profiling of the 3 cathepsins was confirmed in blood cells of an extra cohort. Furthermore, in high glucose (HG)-treated retinal vascular endothelial cell cultures recapitulating the cathepsin expression patterns, Cathepsin B or D downregulation mediated the HG-induced anti-autophagic and pro-apoptotic effects, thereby may contribute to vascular lesions under hyperglycemia. This study demonstrates previously undescribed expression patterns of cathepsins, reveals a novel cathepsin-involved pathogenic mechanism under PDR, and sheds light on potential therapeutic targets to this debilitating retinal disease.

## INTRODUCTION

Diabetes mellitus (DM) is a lifelong metabolic disease hallmarked by hyperglycemia [1]. Proliferative diabetic retinopathy (PDR) is a severe complication of DM that causes vision loss [2]. Hyperglycemia generates an adverse microenvironment in the eye, including oxidative stress [3], accumulation of advanced glycosylation end products [4], dysregulated polyol metabolism [5], production and release of inflammaotry factors [6], and over activation of protein kinase C pathway [7], which may lead to degenerative lesions in both retinal microvessels and neuroretina, retinal ischemia, hypoxia, neovascularization, and eventually PDR [8]. However, the available therapeutic modalities to PDR either generate disappointing efficacies or incur severe side effects. For instance, laser photocoagulation curbs retinal neovascularization, yet laser spots damage neuroretina and impede maintenance and recovery of visual functions [9]. Intravitreal administration of corticosteroids attenuates edema and neovascularization in retina [10, 11], however, it may elicit the adverse effects such as cataract and elevated intraocular pressure [12]. Vitrectomy is more invasive, and the postoperative complications hamper long-term recovery of visual acuity [13]. In addition, the anti-vascular endothelial growth factor (VEGF) drugs only have 50% response rate in the receivers, and long-term blockade of VEGF signaling increases the risk of degeneration in retinal vessels and neurons [14]. Therefore, an effective and safe therapeutic modality to PDR is lacking, for which the primary reason is because the pathogenic mechanisms underlying this debilitating disease remain elusive. The mechanisms underlying PDR should then be discerned to provide leads for improving the therapeutic strategies for the disease.

Proteomics utilizes high-throughput technology to study structure and function of proteins, which facilitates understanding of pathogenesis and progression of disease at protein level [15]. Tandem mass tag (TMT) is one of the common quantitative methods of proteomics [16, 17]. Compared with the traditional gel-based proteomic quantification approach, TMT has better reliability, accuracy, and coverage. In this study, TMT labeling and liquid chromatography and tandem mass spectrometry (LC-MS/MS) were used to identify the differentially expressed proteins in the vitreous humor of the patients with rhegmatogenous retinal detachment (RRD, serving as the control group) and PDR. The proteomic results revealed that the protein expression of Cathepsin B (CTSB), Cathepsin D (CTSD), and Cathepsin B (CTSL) was significantly downregulated in PDR group as compared to RRD group, which was verified at protein and transcript levels in the vitreous and blood samples of additional cohorts. The CTSB, CTSD, and CTSL belong to cathepsin superfamily [18] and are critical lysosomal proteases involved in the fundamental cellular processes, such as intracellular proteolysis [19], extracellular matrix degradation [20], and autophagy [21]. Autophagy is a stringently regulated process in which cellular debris, degenerated organelles, and aged proteins were wrapped by autophagosome and subsequently degraded by fusion of the autophagosome with lysosome [22]. Furthermore, a delicate balance usually exists between autophagy and apoptosis within the cell [23, 24]. Therefore, we hypothesize that the downregulated expression of cathepsin proteins under PDR may reduce autophagic flux and promote apoptosis in the cell. Then a high glucose (HG)-stimulated retinal vascular endothelial cell model was established to recapitulate the PDR condition and test the hypothesis.

## RESULTS

### Screening of differentially expressed proteins in vitreous of RRD and PDR patients

**Figure 1:**
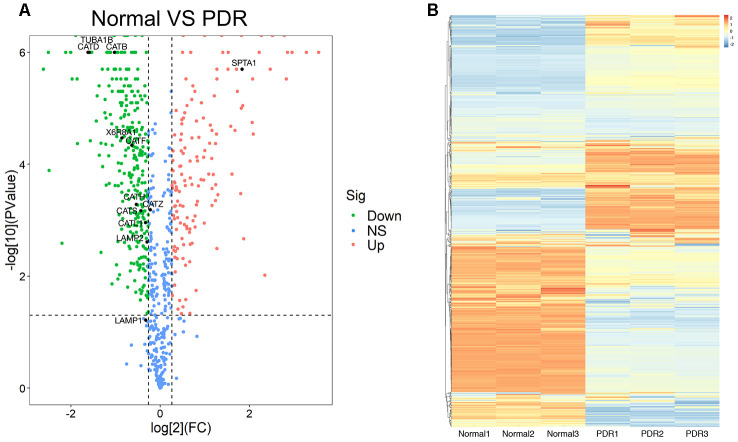
**The proteomics analysis of vitreous samples of the patients with RRD and PDR.** (**A**) A volcano map showed the differentially expressed proteins in vitreous samples of RRD and PDR groups (n = 3/group). The blue dots indicate the proteins whose expression was not significantly different between the two groups; the red dots indicate the significantly upregulated proteins in PDR group as compared to RRD group; the green dots refer to the significantly downregulated proteins. The significantly downregulated CTSB, CTSD, and CTSL proteins were designated. (**B**) Clustering analysis of protein expression in the experimental groups. The color scale on the right illustrated the relative expression levels of proteins in RRD and PDR groups. Red denoted the relative expression level greater than 0 and blue less than 0. RRD: rhegmatogenous retinal detachment; PDR: proliferative diabetic retinopathy; FC: fold change; Sig: significance; Down: downregulation; NS: not significant; Up: upregulation.

A total of 873 proteins were detected in the vitreous samples of RRD group and PDR group. The volcano plot showed that as compared to RRD vitreous fluid, 453 differentially expressed proteins were identified in PDR group. Among the differentially expressed proteins, 145 were significantly upregulated and 308 downregulated. The downregulated proteins included CTSB, CTSD, CTSL, and lysosome-associated membrane glycoprotein 2 (Figure 1A). The top 20 differentially expressed proteins between the two groups were selected according to the *P* value in statistics and the fold change (FC) of the PDR group over RRD group (Table 1). The hierarchical clustering analysis illustrated the distribution of the differentially expressed proteins in the vitreous and distinguished the protein expression profiling between RRD and PDR groups (Figure 1B).

**Table 1:** The top 20 differentially expressed proteins between RRD and PDR groups.

**Gene**	**Description**	**Regulated**	**Fold change (PDR/RRD)**
HBD	Hemoglobin subunit delta	up	7.24
HBB	Hemoglobin subunit beta	up	6.88
S100A6	Protein S100-A6	up	5.73
CAT	Catalase	up	4.81
AK1	Adenylate kinase isoenzyme 1	up	3.53
DMKN	Dermokine	up	2.60
ITIH3	Inter-alpha-trypsin inhibitor heavy chain H3	up	1.99
LYZ	Lysozyme C	up	1.43
ITIH4	Inter-alpha-trypsin inhibitor heavy chain H4	up	1.42
COL6A1	Collagen alpha-1(VI) chain	down	0.76
C1QC	Complement C1q subcomponent subunit C	down	0.68
PRKCSH	Glucosidase 2 subunit beta	down	0.60
TNC	Tenascin	down	0.59
COL9A2	Collagen alpha-2(IX) chain	down	0.59
ASAH1	Acid ceramidase	down	0.58
GAA	Lysosomal alpha-glucosidase	down	0.58
NBL1	Neuroblastoma suppressor of tumorigenicity 1	down	0.49
FCGBP	IgGFc-binding protein OS=Homo sapiens GN	down	0.49
RBP3	Retinol-binding protein 3	down	0.46
SHISA5	Protein shisa-5	down	0.27

### Gene Ontology and KEGG analyses of the differentially expressed proteins

**Figure 2:**
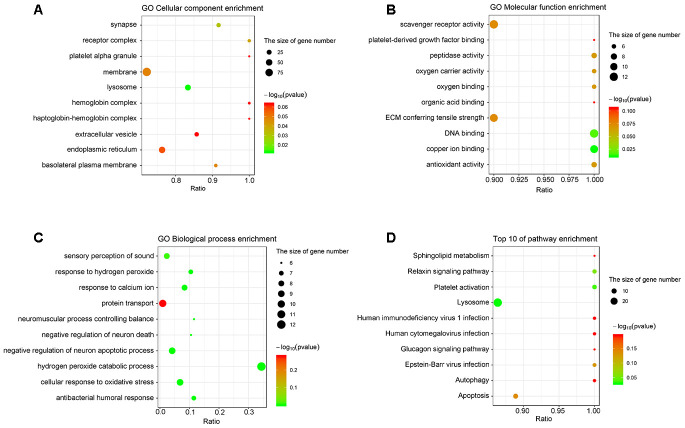
**The analyses of GO enrichment and KEGG pathway.** (**A**–**C**) The Go enrichment analysis revealed that the differentially expressed proteins were enriched in cellular component, molecular function, and biological process. (**D**) KEGG pathway analysis indicated the top 10 signaling pathways enriched by the differentially expressed proteins. The x-axis represents the ratio of candidate genes in the background gene. The y-axis represents the GO terms. The color of the bubble corresponds to the enrichment (-log_10_ (*P* value)), and the size of the bubble is proportional to the number of genes enriched in the pathway.

Gene Ontology (Go) analysis indicated that the cellular components of the differentially expressed proteins involved receptor complex, lysosome, and basolateral plasma membrane (Figure 2A). The molecular functions of the differentially expressed proteins included oxygen carrier activity, oxygen binding, DNA binding, and antioxidant activity (Figure 2B). Moreover, these proteins were enriched in the biological processes, such as sensory perception of sound, response to hydrogen peroxide, response to calcium ion, protein transport, and neuromuscular process controlling balance (Figure 2C).

The results of Kyoto Encyclopedia of Genes and Genomes (KEGG) analysis indicated that the differentially expressed proteins were mainly enriched in the pathways controlling sphingolipid metabolism, relaxin signaling, platelet activation, lysosome function, autophagy, and apoptosis (Figure 2D).

### Protein-protein interactions analysis of the differentially expressed proteins

**Figure 3:**
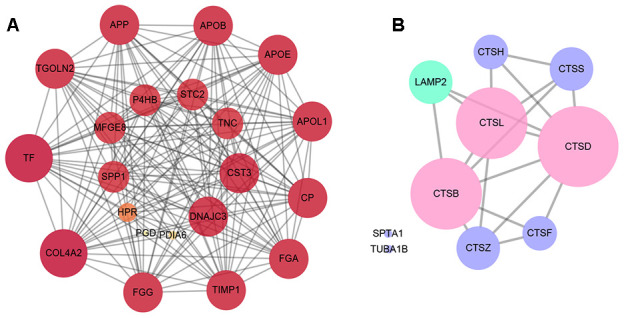
**The analysis of protein-protein interactions.** (**A**) The protein-protein interactions network of the top 20 differentially expressed proteins in the closest connection. (**B**) The protein-protein interactions network of the differentially expressed proteins enriched in autophagy and apoptosis pathways. The green circles refer to the differentially expressed proteins enriched in autophagy pathway, the purple circles represent the proteins enriched in apoptosis pathway, and the pink circles the both.

The Protein-Protein Interactions (PPI) network of the differentially expressed proteins was depicted by Cytoscape software, which was comprised of 333 nodes and 2,808 edges between RRD group and PDR group. The 20 differentially expressed proteins that were in closest connection included trans-Golgi network integral membrane protein 2, protein disulfide-isomerase A1, apolipoprotein L1, ceruloplasmin, fibrinogen gamma chain, metalloproteinase inhibitor 1, and so on (Figure 3A). Interestingly, the PPI network of the proteins enriched in autophagy or apoptosis pathways revealed that CTSB, CTSD, and CTSL, all exhibiting significant downregulation in the PDR patients in comparison to the RRD patients (Table 2), were intimately related to both autophagic and apoptotic pathways (Figure 3B).

**Table 2:** Differential expression of cathepsins in PDR and RRD groups.

**Gene**	**Description**	**Regulated**	**Fold change (PDR/RRD)**	**P value**
CTSB	Cathepsin B	down	0.49	< 0.001
CTSD	Cathepsin D	down	0.33	< 0.001
CTSL	Cathepsin L1	down	0.80	= 0.001

### The expression of CTSB, CTSD, and CTSL in human vitreous humor and peripheral blood

**Figure 4:**
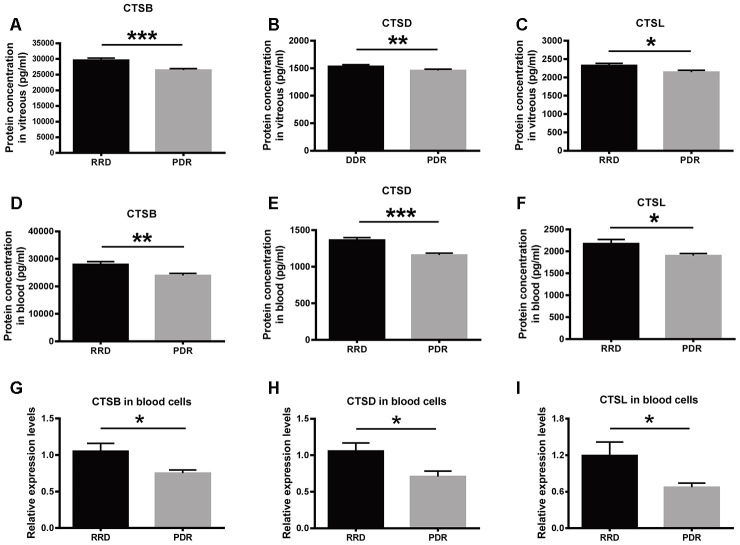
**The expression of CTSB, CTSD, and CTSL in vitreous, serum, and blood cells of patient cohorts.** (**A**–**C**) The protein levels of CTSB, CTSD, and CTSL in vitreous humor of the patients with RRD and PDR. n = 35 – 39 / group. (**D**–**F**) The protein levels of CTSB, CTSD, and CTSL in serum of the patients with RRD and PDR. n = 35 – 39 / group. (**G**–**I**) The relative expression levels of *CTSB*, *CTSD*, and *CTSL* genes in blood cells of an extra patient cohort. n = 12 / group. Data are expressed as mean ± SEM. * *P* < 0.05, ** *P* < 0.01, *** *P* < 0.001.

The expression of the lysosome proteases was verified in vitreous humor and peripheral blood samples collected from RRD and PDR patients. The enzyme-linked immunosorbent assay (ELISA) results showed that the protein expression levels of CTSB, CTSD, and CTSL in the RRD vitreous were 29601.43 ± 4025.79 pg/ml, 1536.13 ± 157.69 pg/ml, and 2325.62 ± 200.99 pg/ml, respectively. The protein levels of the proteases in the PDR vitreous were 26397.95 ± 3355.66 pg/ml, 1451.09 ± 137.82 pg/ml, and 2142.61 ± 130.55 pg/ml, respectively, all of which were significantly lower than the corresponding levels in the RRD group. (Figure 4A–4C, RRD vs PDR, *P* < 0.001 for CTSB, *P* < 0.01 for CTSD; *P* < 0.05 for CTSL). Moreover, the protein expression levels of CTSB, CTSD, and CTSL in the serum of the PDR patients were 23987.04 ± 3660.33 pg/ml, 1159.58 ± 149.55 pg/ml, and 1902.97 ± 197.19 pg/ml, respectively. By contrast, the levels of the 3 enzymes in the RRD serum were 27950.00 ± 4998.11 pg/ml, 1363.05 ± 190.55 pg/ml, and 2174.20 ± 409.34 pg/ml, respectively (Figure 4D–4F). Consistent with the trend in the vitreous, the protein levels of the 3 lysosomal proteases in the serum were all significantly reduced in the PDR patients as compared to the RRD patients (RRD vs PDR, *P* < 0.01 for CTSB, *P* < 0.001 for CTSD, *P* < 0.05 for CTSL). These results confirmed the downregulated protein expression of the 3 enzymes under PDR condition.

At the transcript level, the qPCR results demonstrated that the abundance of *CTSB*, *CTSD* and *CTSL* transcripts in peripheral blood cells were significantly decreased in the PDR group, as compared to that in the RRD group (Figure 4G–4I, RRD vs PDR, all *P* < 0.05), indicating that the expression of these 3 genes were negatively regulated at the transcription level in PDR patients.

### The expression of CTSB, CTSD, and CTSL in simian retinal vascular endothelial cells stimulated with high glucose

**Figure 5:**
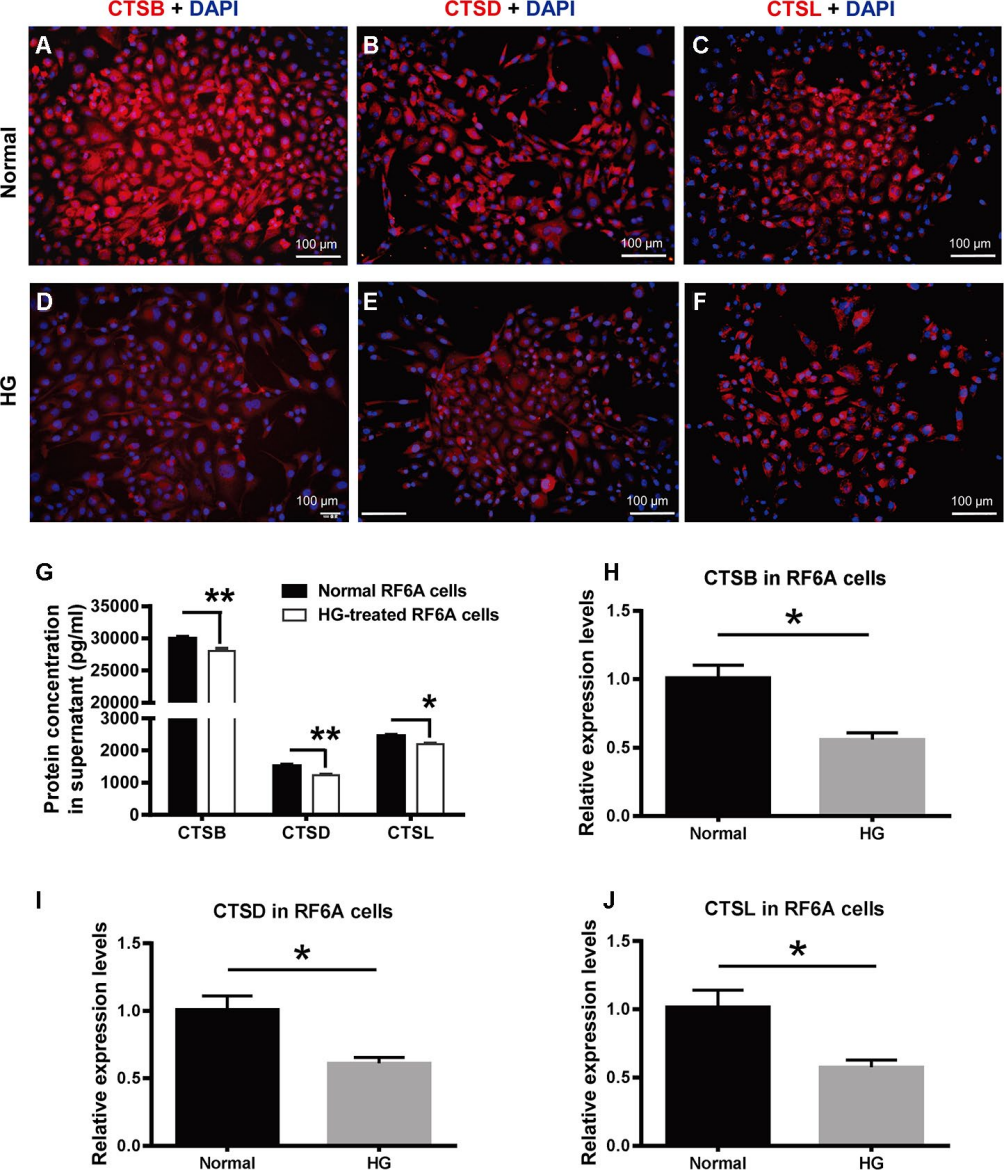
**The expression of CTSB, CTSD, and CTSL in retinal vascular endothelial cells treated with high glucose.** (**A**–**F**) The immunofluorescence of CTSB, CTSD, and CTSL proteins in the HG-treated simian retinal vascular endothelial cells (RF/6A) and normal controls. Scale bar = 100 μm (**G**) The concentrations of CTSB, CTSD, and CTSL proteins in RF/6A cell culture supernatant. (**H**–**J**) The relative expression levels of CTSB, CTSD, and CTSL genes in HG-stimulated and normal RF/6A cells. n = 6 / group. Data are expressed as mean ± SEM. * *P* < 0.05, ** *P* < 0.01. HG: high glucose.

To further understand the cellular consequence of the downregulated cathepsin expression under PDR, a simian retinal vascular endothelial cell line (RF/6A) was treated with HG (25 mM) for a relatively long time (48 h) to simulate the microenvironment under PDR. The immunofluorescence was used to detect CTSB, CTSD, and CTSL protein expression in the retinal vascular endothelial cells. The results showed that these proteins were localized in the cytoplasm. Under normal condition, the fluorescence intensity of CTSB appeared to be greater than those of CTSD and CTDL (Figure 5A–5C), which was similar to the protein expression tendency exhibited in human vitreous and serum samples (Figure 4A–4F). Additionally, the fluorescence intensities of CTSB, CTSD, and CTSL were all seemed subdued in the HG-treated endothelial cells as compared to those in the normal controls (Figure 5).

Moreover, ELISA results showed that the concentrations of the secreted CTSB, CTSD, and CTSL in RF/6A cell supernatant were 30006.67 ± 590.02 pg/ml, 1520.80 ± 93.72 pg/ml, and 2460.37 ± 79.62 pg/ml in the normal control group, and were 28076.67 ± 697.59 pg/ml, 1236.37 ± 58.53 pg/ml, and 2201.93 ± 51.48 pg/ml in the HG group (Figure 5G). The concentrations of the 3 secreted proteins were significantly decreased in the HG group as compared with those in the normal control group (Figure 5G, Normal vs HG, *P* < 0.01 for CTSB, *P* < 0.01 for CTSD, *P* < 0.05 for CTSL). The cathepsin concentrations in RF/6A cell supernatant were comparable to those in human vitreous and serum, and verified the downregulated expression pattern of CTSB, CTSD, and CTSL in the HG-treated cultures as indicated by immunofluorescence (Figure 5A–5F).

Parallel to the gene expression profiling in human blood cells, the expression of *CTSB*, *CTSD,* and *CTSL* genes in HG-treated RF/6A cells was all significantly downregulated as compared to the normal control cells (Figure 5H–5J; Normal vs HG, all *P* < 0.05). These results implicate that the relatively long-term HG treatment (48 h) of retinal vascular endothelial cells recapitulates, at least from the gene expression perspective, the pathological condition of PDR.

### HG stimulation suppressed autophagy and promoted apoptosis in retinal vascular endothelial cells

**Figure 6:**
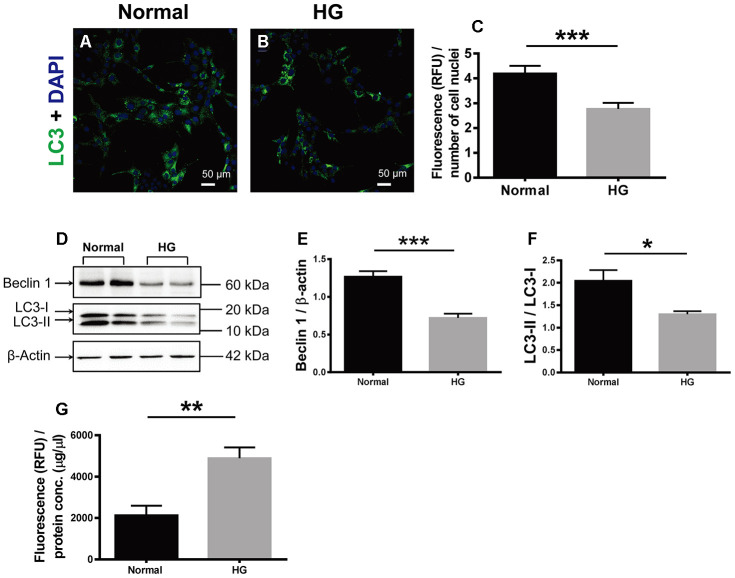
**The effects of high glucose on autophagy and apoptosis of retinal vascular endothelial cells.** (**A**, **B**) Immunofluorescence of LC3, an autophagosomal marker, in HG-treated RF/6A cells and normal controls. Scale bar = 50 μm (**C**) The fluorescence intensity of LC3 was quantified and normalized to the number of cell nuclei. (**D**) Western blot representatives of Beclin1, LC3-I, LC3-II, and β-actin. (**E**) Quantification of Beclin 1 protein levels in the RF/6A cells treated with HG-containing or normal culture media. (**F**) Ratio of LC3-II over LC3-I in the HG-treated RF/6A cells and normal controls. (**G**) The Caspase-3/7 activity (RFU) in the retinal vascular endothelial cells was quantified and normalized to the total protein concentration (μg/μl). n = 6 / group. Data are expressed as mean ± SEM. * *P* < 0.05, ** *P* < 0.01, *** *P* < 0.001.

The effects of HG on cell autophagy and apoptosis were examined. During autophagy, LC3 is the only protein component on the inner membrane of autophagosome. LC3 exists in two forms, cytosolic LC3-I and membrane-bound LC3-II [22]. The ratio of LC3-I conversion to LC3-II has been used as the autophagosomal marker in mammalian cells [25, 26]. Therefore, LC3 immunostaining was performed in the retinal vascular endothelial cells. The positive LC3 signals appeared punctate and located in the cytoplasm. The fluorescence intensity of LC3 signal, indicative of autophagosome formation activity, in the HG-treated cells was 66.67% of that in the normal controls (Figure 6A; Normal vs HG, *P* < 0.001). Consistently, the ratio of LC3-II over LC3-I (LC3-II / LC3-I) in the HG group was 63.54% of the normal controls (Figure 6D, 6F; Normal vs HG, *P* < 0.05). In addition, the abundance of Beclin 1, another positive regulator of autophagy [22], in the HG-treated cells was reduced to 56.90% of the normal level (Figure 6D, 6E; Normal vs HG, *P* < 0.001). By contrast, the apoptotic activity, as reflected by the activity of caspase-3/7, the ultimate executor of apoptosis [27], was boosted 2.29 fold in the HG group as compared to the normal control group (Figure 6G, Normal vs HG, *P* < 0.01). These results suggest that autophagy was significantly suppressed and apoptosis promoted in the retinal vascular endothelial cells treated with HG for a relatively long time.

### The inhibitors or overexpression of cathepsins mimicked or abrogated the effects of HG in the retinal vascular endothelial cells

In view of the facts that CTSB, CTSD, and CTSL are enriched in both autophagy and apoptosis pathways (Figure 3), the expression of the 3 proteins was all downregulated in HG-treated cells (Figure 5), and HG stimulation resulted in waned autophagy and augmented apoptosis (Figure 6), we tried to ascertain whether the effects of HG on the endothelial cells were through downregulated expression of cathepsins. According to the results of proteomics and ELISA, the downregulation of CTSB and CTSD proteins was more significant than CTSL in both human and cell samples (Table 2 and Figures 4, 5), hence the roles of CTSB and CTSD were preferentially tested.

**Figure 7:**
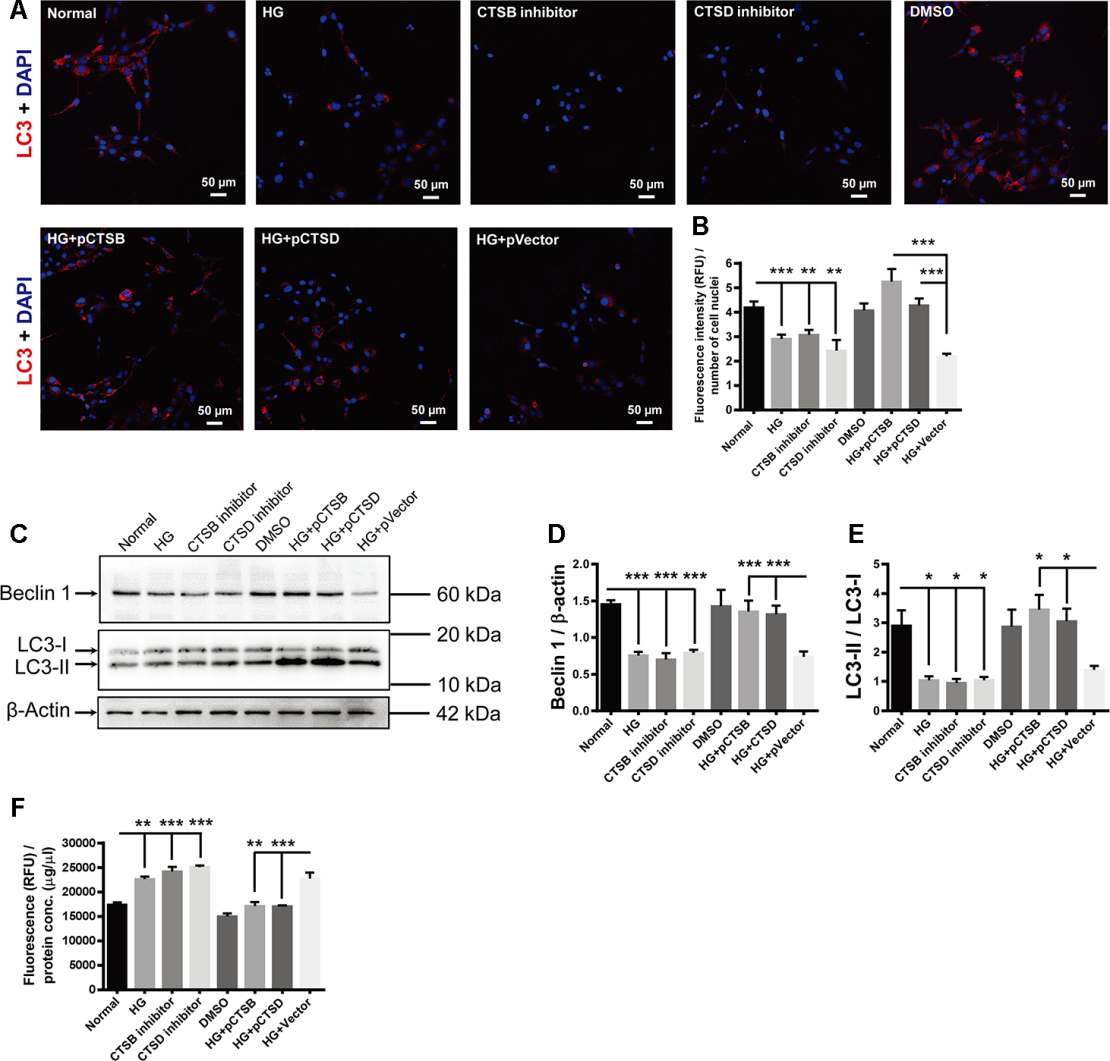
**The effects of high glucose on autophagy and apoptosis were mimicked by CTSB and CTSD inhibitors and abrogated by their overexpression.** (**A**) Immunofluorescence staining of LC3 in experimental groups. Scale bar = 50 μm (**B**) The fluorescence intensity of LC3 was quantified and normalized to the number of cell nuclei. (**C**) The representative pictures of Western blots of Beclin1, LC3-I, LC3-II, and β-actin. (**D**, **E**) Quantification of Beclin 1 protein levels and the ratio of LC3-II over LC3-I in the HG-treated RF/6A cells and normal controls. (**F**) The Caspase-3/7 activity (RFU) in the retinal vascular endothelial cells was quantified and normalized to the total protein concentration (μg/μl). n = 6 / group. Data are expressed as mean ± SEM. * *P* < 0.05, ** *P* < 0.01, *** *P* < 0.001.

The red fluorophore was used for LC3 staining in this experiment to distinguish the fluorescence from the green fluorescence protein (GFP) carried by the transfected recombinant plasmids. The fluorescence intensity of LC3 punctate signals was significantly reduced in the HG-treated cells as compared to the normal controls (Figure 7A, 7B; Normal vs HG, *P* < 0.001). This reduction was mimicked by incubating the normal control cells with specific inhibitors of CTSB or CTSD (Figure 7A, 7B; both *P* < 0.01, for Normal vs CTSB inhibitor and Normal vs CTSD inhibitor). On the contrary, transfection of CTSB- or CTSD-encoding plasmids, but not empty vector, abrogated the HG-induced reduction in LC3 staining intensity (Figure 7A, 7B; both *P* < 0.001, for HG + pCTSB vs HG + pVector and HG + pCTSD vs HG + pVector; *P* > 0.05, for HG vs HG + pVector). Likewise, the abundance of Beclin 1 and LC3-II / LC3-I exhibited the similar trends to that of LC3 staining among experimental groups (Figure 7C–7E; all *P* < 0.001 for Beclin 1, all *P* < 0.05 for LC3-II / LC3-I, Normal vs HG, Normal vs CTSB inhibitor, Normal vs CTSD inhibitor, HG + pCTSB vs HG + p Vector, HG + pCTSD vs HG + pVector; *P* > 0.05, for HG vs HG + pVector). On the other hand, the apoptotic activity was boosted by the treatments of HG and cathepsin inhibitors, but not by the solvent control DMSO (Figure 7F; *P* < 0.01, Normal vs HG; both *P* < 0.001, for Normal vs CTSB inhibitor and Normal vs CTSD inhibitor; *P* > 0.05, for Normal vs DMSO). Under stimulation of HG, the apoptotic activity was subdued by overexpressing CTSB or CTSD, but not by overexpressing the empty vector (Figure 7F; *P* < 0.01, for HG + pCTSB vs HG + pVector; *P* < 0.001, for HG + pCTSD vs HG + pVector; *P* > 0.05, for HG vs HG + pVector). These results implicated that downregulating expression of cathepsins (CTSB and CTSD) or inhibiting their activities was both sufficient and necessary to mediate the HG-induced autophagic suppression and apoptotic promotion.

## DISCUSSION

In the current study, the TMT labeling and LC-MS/MS techniques were used to identify the differentially expressed proteins in the vitreous humor between RRD and PDR patients. The proteomic results revealed that as compared with the RRD control group, the lysosomal proteases CTSB, CTSD, and CTSL in the PDR group were significantly downregulated at the protein levels (Figure 1A and Table 2). This trend of reduction was validated by ELISAs in the vitreous humor and serum of another cohort (Figure 4A–4F). Moreover, the downregulated expression of *CTSB*, CTSD, and *CTSL* genes at mRNA level was confirmed in the blood cells of an extra cohort (Figure 4G–4I). These results indicate that the expression patterns of the 3 cathepsin genes were similar in the circulatory system and may be regulated at the transcription level.

To our knowledge, we, for the first time, report the downregulation of CTSB at both protein and transcript levels in PDR patients as compared to RRD patients. What’s more, CTSB protein, at the basal level, was a thousand fold more abundant than CTSD and CTSL proteins in human vitreous and serum (Figure 4A–4F), implicating that CTSB might be the predominant form of cathepsin in human circulation. On the other hand, the downregulated expression of CTSL under PDR was consistent with the previous findings from diabetic patients. For example, the *CTSL* transcript levels in muscle biopsies of type 2 diabetic patients following insulin infusion were significantly lower than those in the healthy subject controls [28]. Additionally, the protein abundance and enzymatic activity of CTSL in endothelial progenitor cells (EPCs) isolated from type 2 diabetic patients were merely 50% of those in the EPCs isolated from health controls [29]. By contrast, an Indian group has reported the upregulated CTSD expression under diabetic condition. They found that CTSD protein levels were significantly boosted in the serum of the young patients (< 25 y) with type 2 diabetes as compared to the subjects with normal glucose tolerance [30]. The discrepancy between their results and ours might be explained by the disparate control groups in the two studies. The Indian researchers recruited the individuals with normal glucose tolerance; whereas we enrolled the RRD patients as controls. Although the RRD patients were non-diabetic based on the inclusion criteria, the RRD might potentially influence the expression of cathepsins, it would be interesting in the future to include age-matched healthy volunteers as another control group, thereby eliminating the effect of RRD as a confounding factor.

The KEGG analysis revealed that CTSB, CTSD, and CTSL were all enriched in and closely related to autophagy and apoptosis pathways (Figure 3B), indicating the possible regulation of the 3 cathepsins on these two fundamental cellular processes. Then a HG-treated retinal vascular endothelial cell model was established to recapitulate the downregulated gene expression pattern of the 3 cathepsins (Figure 5). Subsequently, the effects of HG on autophagy and apoptosis were examined in the cell culture model. The averaged LC3 immunofluorescence intensity reflecting the number and forming capacity of autophagosomes, the conversion ratio of LC3-I to LC3-II, and Beclin 1 serving as a critical regulator of autophagosome generation were used as surrogates for autophagic activity (Figure 6A–6F). Indeed, all the 3 surrogates were significantly suppressed in retinal vascular endothelial cells under HG stimulation (Figure 6A–6F), in parallel to the downregulated cathepsin expression (Figure 5). On the contrary, the apoptotic activity of the cells, indicated by Caspase-3/7 activity, was significantly enhanced (Figure 6G).

Then the next question would be whether the anti-autophagic and pro-apoptotic effects of HG were mediated by the downregulation of cathepsin expression. To address this question, loss of function of CTSB and CTSD was achieved by application of specific pharmacological inhibitors to the normal cell cultures; whereas gain of function of CTSB and CTSD was achieved by overexpression of the plasmids encoding each lysosome protease. The results demonstrated that the HG-induced suppression of autophagy and promotion of apoptosis in retinal vascular endothelial cells was mimicked by incubating the normal cells with the inhibitor of either cathepsin (Figure 7); whereas the effects of HG were abolished by overexpression of either CTSB or CTSD (Figure 7). These results suggest that the HG’s anti-autophagic and pro-apoptotic effects in retinal vascular endothelial cells were mediated by downregulated expression of CTSB or CTSD, implicating the novel therapeutic targets to the vessel lesions of PDR.

There are limitations in this study. First, although the identification and validation of the differentially expressed proteins were conducted in 3 different cohorts, the sizes of the cohorts were relatively small, it would be interesting to expand the cohorts and include a healthy control group as mentioned above in the future study, aiming to corroborate the gene expression pattern and optimize the therapeutic target. Second, we have shown that HG generates opposite effects on autophagy and apoptosis in our culture paradigm, nonetheless, the molecular pathways linking the two processes remain unclear. Finally, albeit functional redundancy might exist between the cathepsins, the cellular consequences of downregulated CTSL expression in our culture paradigm warrant investigation.

In summary, TMT-labeled LC-MS/MS revealed that the CTSB, CTSD, and CTSL were significantly downregulated in the vitreous of PDR group as compared to RRD group and were enriched in autophagy and apoptosis pathways. The expression of the 3 cathepsins was verified at the protein and transcript levels in vitreous, serum, and blood cells of additional cohorts. In the HG-treated retinal vascular endothelial cell model recapitulating PDR condition, downregulated CTSB or CTSD suppressed autophagy and promoted apoptosis, thereby contributing to the vascular pathology of PDR.

## MATERIALS AND METHODS

### Subjects and sample collection

The subjects of this study were recruited from the patients admitted to the Department of Fundus and Ocular Trauma in Tianjin Medical University Eye Hospital from February, 2018 to February, 2019. The patients were diagnosed with RRD according to the published international standards [31]; whereas the PDR patients were diagnosed based on the guidelines issued on the website of the American Academy of Ophthalmology (https://www.aao.org/topic-detail/diabetic-retinopathy-europe). Both groups received transciliary vitrectomy. The patients who had other ocular disorders, such as trauma, glaucoma, uveitis, and serious vitreous hemorrhage, affecting general conditions of the eye or the patients with a history of severe systemic diseases, including heart failure, renal failure, hematological and cardiovascular diseases, were excluded from the study. Moreover, the patients who had received retinal laser photocoagulation or anti-VEGF treatment or had a history of eye surgery were also excluded. All the recruited patients provided written informed consent prior to inclusion.

Vitreous humor samples were collected during vitrectomy from the patients with RRD (control group) or PDR (PDR group). The samples were snap frozen in liquid nitrogen and stored at -80° C. The blood samples were collected in an anti-coagulation tube during conventional tests upon admission. The demographic information of the patients whose samples were used for analyses of cathepsin expression at the protein level was listed in Table 3; whereas that of the patients recruited for analyzing cathepsin expression at the transcript level was shown in Table 4. All procedures of sample collection were approved by the Ethics Committee and the Institutional Review Committee of Tianjin Medical University Eye Hospital and were in accordance with the tenets of the Declaration of Helsinki.

**Table 3:** Demographic information of the patients for cathepsin protein expression analyses.

**Groups**	**Number**	**Gender (M/F)**	**Age (y)**	**Diagnosis**
Control	#1	M	39	RRD
	#2	F	43	RRD
	#3	F	69	RRD
	#4	M	55	RRD
	#5	F	62	RRD
	#6	F	43	RRD
	#7	M	74	RRD
	#8	M	55	RRD
	#9	M	74	RRD
	#10	F	67	RRD
	#11	M	78	RRD
	#12	F	68	RRD
	#13	M	37	RRD
	#14	M	68	RRD
	#15	M	53	RRD
	#16	F	53	RRD
	#17	F	58	RRD
	#18	F	56	RRD
	#19	M	60	RRD
	#20	M	63	RRD
	#21	M	73	RRD
	#22	F	41	RRD
	#23	F	59	RRD
	#24	F	60	RRD
	#25	M	47	RRD
	#26	F	68	RRD
	#27	F	56	RRD
	#28	M	62	RRD
	#29	F	50	RRD
	#30	F	53	RRD
	#31	M	59	RRD
	#32	F	65	RRD
	#33	F	70	RRD
	#34	F	46	RRD
	#35	M	52	RRD
PDR	#1	F	50	PDR
	#2	F	40	PDR
	#3	F	59	PDR
	#4	F	49	PDR
	#5	F	30	PDR
	#6	M	35	PDR
	#7	M	55	PDR
	#8	F	58	PDR
	#9	M	54	PDR
	#10	F	58	PDR
	#11	F	45	PDR
	#12	M	53	PDR
	#13	F	50	PDR
	#14	F	67	PDR
	#15	M	53	PDR
	#16	F	63	PDR
	#17	F	66	PDR
	#18	M	71	PDR
	#19	F	63	PDR
	#20	M	50	PDR
	#21	F	55	PDR
	#22	F	69	PDR
	#23	F	56	PDR
	#24	F	71	PDR
	#25	M	52	PDR
	#26	F	62	PDR
	#27	M	61	PDR
	#28	M	50	PDR
	#29	M	71	PDR
	#30	M	72	PDR
	#31	F	63	PDR
	#32	F	63	PDR
	#33	F	71	PDR
	#34	F	62	PDR
	#35	F	72	PDR
	#36	F	66	PDR
	#37	F	65	PDR
	#38	F	67	PDR
	#39	F	64	PDR

Note: RRD: rhegmatogenous retinal detachment; PDR: proliferative diabetic retinopathy; F: female; M: male.

**Table 4:** Demographic information of the patients for cathepsin gene expression analysis.

**Groups**	**Number**	**Gender (M/F)**	**Age (y)**	**Diagnosis**
Control	#1	M	74	RRD
	#2	M	53	RRD
	#3	F	64	RRD
	#4	F	70	RRD
	#5	M	68	RRD
	#6	M	64	RRD
	#7	F	61	RRD
	#8	F	62	RRD
	#9	M	67	RRD
	#10	F	61	RRD
	#11	F	72	RRD
	#12	F	60	RRD
PDR	#1	M	61	PDR
	#2	M	73	PDR
	#3	M	67	PDR
	#4	M	61	PDR
	#5	F	59	PDR
	#6	F	54	PDR
	#7	F	64	PDR
	#8	F	53	PDR
	#9	M	54	PDR
	#10	M	74	PDR
	#11	F	42	PDR
	#12	M	47	PDR

Note: RRD: rhegmatogenous retinal detachment; PDR: proliferative diabetic retinopathy; F: female; M: male.

### Measurement of total protein concentrations

Total protein concentrations of the samples were determined using a Bicinchoninic Acid (BCA) Protein Assay Kit (CWBIO, Beijing, China). Bovine serum albumin (BSA) standard (2 mg / ml, CWBIO, Beijing, China) was serially diluted to generate a standard curve and served as positive controls for protein quantification. The diluent was included as a negative control. Then 25 μl of the standards and the appropriately diluted samples were incubated with the BCA reagents at 37° C for 30 min. The absorbance at 562 nm was measured. The protein concentration (μg/μl) of each sample was read off the standard curve and multiplied by the dilution factor.

### Filter-aided sample preparation and tandem mass tag labeling

The preparation and labeling of the RRD and PDR samples (n = 3/group) were performed using reagents from a TMT duplex Isobaric Labeling Reagent Set (Thermo Fisher Scientific, Waltham, MA, USA) according to the company’s protocol. Briefly, 100 μg of total protein from each sample were incubated with 5 μl reducing reagent (Thermo Fisher Scientific, Waltham, MA, USA) at 55° C for 1 h. Another 5 μl iodoacetamide solution (Thermo Fisher Scientific, Waltham, MA, USA) was added and incubated with the reaction mixture for 30 min in the dark at room temperature (RT). Then 200 μl of dissolution buffer (Thermo Fisher Scientific, Waltham, MA, USA) was added and the resulting mixture was transferred to a Microcon-10 kDa Centrifugal Filter Unit with Ultracel-10 membrane (Millipore, Danvers, MA, USA) and centrifuged at 12,000 rpm for 20 min, the filtrate was discarded. The centrifugation was repeated 4 times. The solution retained in the upper chamber contained the enriched proteins with molecular weights higher than 10 kDa, which were digested by trypsin at 37° C overnight. Next day, the digested proteins were washed 3 times with ultrapure water (resistivity > 18.2 ΩM.cm), lyophilized, and dissolved in 100 mM dissolution buffer (Thermo Fisher Scientific, Waltham, MA, USA). Subsequently, 100 μg digested proteins (~ 1 μg/μl) were incubated at RT for 1 h with the TMT reagent (Thermo Fisher Scientific, Waltham, MA, USA) reconstituted with anhydrous ethanol (Sigma-Aldrich, St. Louis, MO, USA). The TMT labeling was terminated by adding a quenching reagent (Thermo Fisher Scientific, Waltham, MA, USA). The labeled protein samples were thoroughly mixed, precipitated, lyophilized, and stored at –80° C for future usage.

### Off-line pre-separation of the TMT-labelled digested peptides and LC-MS/MS

The TMT-labelled lyophilized protein samples were dissolved in 100 μl flow phase A solution composed of 98% double distilled water and 2% acetonitrile (pH 10) (Merck, Darmstadt, Germany), centrifuged at 12,000 rpm for 20 min, and the supernatant was collected. The affinity chromatography was pre-run by isolating 400 μg of trypsin-digested BSA, the temperature was set at 45° C, and the detection wavelength at 214 nm. Afterwards, 100 μl of the trypsin-digested and TMT-labelled samples were loaded to the column, the flow rate was 0.7 ml/min. Then the isolated components were re-dissolved in 20 μl solvent comprised of 2% methanol and 0.1% formic acid, and centrifuged at 12,000 rpm for 10 min. Ten microliters of the supernatant were used for LC-MS/MS.

### Analysis of differentially expressed proteins

The FC was reported as the ratio of the abundance of a particular protein in the PDR group over its abundance in the RRD group. The differentially expressed proteins were screened out using the filtering criteria as *P* value < 0.05 and FC > 1.20 or < 0.83. Moreover, GO enrichment, KEGG pathway, and PPI network were analyzed. The GO enrichment of differentially expressed proteins were determined by DAVID 6.8 (http://david-d.ncifcrf.gov) software. The PPI network was constructed by introducing the information from the Search Tool for the Retrieval of Interacting genes (STRING) database into Cytoscape (3.5.1. version) software and delineating the interactive network. The mass spectrometry proteomics data have been deposited to the ProteomeXchange Consortium via the PRIDE [32] partner repository with the dataset identifier PXD021788.

### Enzyme-linked immunosorbent assay

The vitreous humor and serum samples from the RRD group (n = 35) and PDR groups (n = 39), as well as the supernatant of the cells treated with normal or HG-containing culture media (n = 6 / group) were collected. The concentrations of Cathepsin B (CTSB), Cathepsin D (CTSD), and Cathepsin L (CTSL) in the samples were measured by ELISA kits (Jinweizhi Biotechnology Co., Ltd, Suzhou, China; for CTSB ELISA kit Catalog#: m1645803; CTSD ELISA kit Catalog#: m1645800; CTSL ELISA kit Catalog#: m1645802). Briefly, the serially diluted standards and appropriately diluted samples were added to a 96-well microplate pre-coated with the primary monoclonal antibodies to the target proteins. Following incubation at RT for 2 h, the microplates were decanted and thoroughly washed. Then 100 μl of HRP-conjugated detection antibodies were added to sandwich the target proteins bound to the primary antibodies. After extensive washes, the chromogenic substrate was added. After incubation at 37° C for 15 min in the dark, the stop solution was added to terminate the reaction. The absorbance at 450 nm was measured by an Infinite 200 PRO Multimode Microplate Reader (Tecan Group Ltd., Männedorf, Switzerland). The concentrations of CTSB, CTSD, and CTSL proteins in each sample were read off the standard curves and multiplied by the dilution factors.

### RNA extraction and reverse transcription

Peripheral blood (2 - 5 ml) samples collected from the RRD or PDR patients were centrifuged at 1,800 g for 5 min. In one batch of samples, the sera were used for ELISA as mentioned above. In another batch of samples, the cell pellets (n = 12 / group) following centrifugation were re-suspended with phosphate-buffered saline (PBS), then erythrocyte lysis buffer (Solarbio, Beijing, China) was added. After another round of centrifugation, the top liquid layer was removed, and the middle white layer containing peripheral blood mononuclear cells (PBMCs) was collected. Total RNA of the PBMCs was extracted using TRIzol reagent (Thermo Fisher Scientific, Waltham, MA, USA). The reverse transcription was performed in a 20-μl-reaction system using 1 μg total RNA and the reagents in 5X All-In-One RT MasterMix (abm, Richmond, BC, Canada) according to manufacturer’s protocols.

### Quantitative real-time PCR

The PCR reaction mixture was composed of cDNA template, forward and reverse primers (Table 5), and SYBR Green FastStart 2X Master Mix (abm, Richmond, BC, Canada). One microliter cDNA from each sample was pooled, serially-diluted, and used as templates to generate a standard curve between Ct values of the gene to be examined and logarithms of cDNA template concentrations. The standard curves demonstrated the similar priming efficiency between each cathepsin gene and *Gapdh* gene. The standard curves also served as positive controls, and the reactions using water as template served as negative controls. The reaction mixture was added in triplicate into a 384-well plate. The qPCR program consisted of pre-incubation at 50° C for 2 min, denaturation at 95° C for 10 min, 40 cycles of denaturation at 95° C for 15 s and extension at 60° C for 1 min. A dissociation stage was added to check amplicon specificity. The amplification was performed in a HT7900 Real-Time PCR System (Applied Biosystem, Foster City, CA, USA). The relative expression levels of the cathepsin genes were analyzed using a comparative threshold cycle (2^−ΔΔCt^).

**Table 5:** Primer sequences.

**Gene**	**species**	**Pubmed ID**	**Purpose**	**Sequence**
*GAPDH*	human	NM_002046.7	qPCR	Forward: 5’-ATGGAAATCCCATCACCATCTT-3’
				Reverse: 5’-CGCCCCACTTGATTTTGG-3’
*CTSB*	human	NM_001908.5	qPCR	Forward: 5’-TGTAATGGTGGCTATCCTGCT-3’
				Reverse: 5’-AGGCTCACAGATCTTGCTACA-3’
*CTSD*	human	NM_001909.5	qPCR	Forward: 5’-CACAAGTTCACGTCCATCCG-3’
				Reverse: 5’-TGCCAATCTCCCCGTAGTAC-3’
*CTSL*	human	NM_001912.5	qPCR	Forward: 5’-GAAGGCGATGCACAACAGAT-3’
				Reverse: 5’-GTCTCCAAAGGCGTTCATGG-3’
*CTSB*	monkey	NM_001194899.2	expression	Forward: 5’-GCCTCGAGGCCACCATGTGGCGGCTCTGGGCCT-3’
				Reverse: 5’-GCGGATCCTTAGATCTTTTCCCAGTA-3’
*CTSD*	monkey	NM_001260554.1	expression	Forward: 5’-GCCTCGAGGCCACCATGCATCCCCCCAGCCTTCT-3’
				Reverse: 5’-GCGGATCCCTAGAGGCGGGCAGCCTC-3’

Note: GAPDH: glyceraldehyde-3-phosphate dehydrogenase; CTSD: Cathepsin D; CTSB: Cathepsin B; CTSL: Cathepsin L.

### Cell cultures

A simian retinal vascular endothelial cell line RF/6A was purchased from the Chinese Academy of Science (Shanghai, China) and maintained in complete culture media composed of RPMI-1640 (Thermo Fisher Scientific, Waltham, MA, USA), 10% fetal bovine serum (Thermo Fisher Scientific, Waltham, MA, USA), 100 U/ml penicillin, 100 μg/ml streptomycin (Thermo Fisher Scientific, Waltham, MA, USA), and 2 mM L-glutamine (Thermo Fisher Scientific, Waltham, MA, USA) under 37° C and 5% CO_2_ in a humidified incubator.

The RF/6A cells at the 3^rd^ passage were seeded in 24-well plates (2X10^5^ / ml, Corning, Corning, NY, USA) or 6-well plates (5X10^5^ / ml, Corning, Corning, NY, USA). The cells were divided into normal control and HG groups (n = 6 / group). During the experiments, the cells in the normal control group were cultured with complete culture media (5 mM glucose); the cells in the HG group were treated by the complete culture media containing 25 mM glucose (Sigma-Aldrich, St. Louis, MO, USA). Forty-eight hours later, the protein expression of CTSB, CTSD, and CTSL in the RF/6A cells and supernatant was analyzed by immunofluorescence and ELISA, respectively. The expression of the 3 genes in the cells was evaluated by qPCR as described above.

### Immunofluorescence

The RF/6A cells were fixed with 4% paraformaldehyde (Solarbio, Beijing, China), washed with PBS, permealized with 0.1% Triton X-100 (Solarbio, Beijing, China), and blocked with 5% BSA (Solarbio, Beijing, China) for 2 h at RT. Then the cells were incubated with the rabbit primary antibodies to CTSB (1:250, Bioss, Beijing, China), CTSD (1:250, Bioss, Beijing, China), and CTSL (1:250, Bioss, Beijing, China) at 4° C overnight. The next day, the cells were washed with PBS, and incubated with the corresponding Alexa 596-conjugated secondary antibodies at RT for 1 h. The cells were subsequently counter stained with 4, 6-diamidino-2-phenylindole (DAPI, Solarbio, Beijing, China) and photographed under a fluorescence microscope (BX51; Olympus Optical Co. Ltd., Tokyo, Japan) using the cellSens Standard electronic system (Olympus Optical Co. Ltd., Tokyo, Japan) with identical optical parameters.

### Molecular cloning of monkey *CTSB* and *CTSD* genes

The sequences encoding monkey CTSB and CTSD were amplified by PCR from the cDNA derived from RF/6A cells cultured with complete culture media using the expression primers listed in Table 5. Each PCR product was cloned into a mammalian expression vector downstream cytomegalovirus promoter and upstream the gene cassette containing the GFP gene driven by internal ribosome entry site (IRES-GFP) through restriction endonuclease sites *XhoI* and *BamHI* using T4 DNA ligase (Thermo Fisher Scientific, Waltham, MA, USA). The Kozak sequence (GCCACC) was inserted in the immediate front of initiation codon (ATG) to enhance expression efficiency. After confirmation by DNA sequencing, the recombinant and empty vectors were purified by EndoFree Plasmid Maxi Kit (QIAGEN China, Shanghai, China) and designated as pCTSB, pCTSD, and pVector, respectively.

### Transfection of retinal microvessel endothelial cells

The RF/6A cells at the 3^rd^ passage were cultured at a density of 4X10^5^ / ml in the 6-well plates that had been coated with rat tail collagen (Sigma-Aldrich, St. Louis, MO, USA). At 90 - 95% confluency, each well of cells were transfected with 2 μg pCTSB or pCTSD or pVector (n = 6 / group) using X-tremeGENE HP DNA Transfection Reagent (Roche, Branford, CT, USA). Twenty-four hours post transfection, the cells were processed for the subsequent experiments.

### Activity of autophagosome formation in retinal vascular endothelial cells

The sterilized cover slips were placed in a 24-well plate and coated with rat tail collagen (Sigma-Aldrich, St. Louis, MO, USA). The RF/6A cells were seeded on the cover slips at the density of 1.6X10^5^ / ml. In one set of experiments, the cells were divided into normal control and HG groups (n = 6 / group), and were treated as described above. In the other set of experiments, the cells were divided into 8 groups, including normal control, HG, CTSB inhibitor, CTSD inhibitor, DMSO, HG + pCTSB, HG + pCTSD, and HG + pVector groups (n = 6/group). The normal and HG groups of cells were treated as mentioned above. Based on the literature, the CTSB inhibitor CA-074 methyl ester at 50 μM (Selleck Chemicals, Houston, TX, USA; stock solution 50 mM in DMSO) [33, 34], the CTSD inhibitor Pepstatin A at 100 μM (Selleck Chemicals, Houston, TX, USA; stock solution 100 mM in DMSO) [35], and 0.1% DMSO were added to the complete culture media of the corresponding groups. In addition, the transfected cells were trypsinized and seeded onto the cover slips and cultured with HG-containing media. Following the 48 h treatments, the cells were subjected to immunofluorescence as described above, except that rabbit monoclonal antibody to LC3A/B (1:100, Cell Signaling Technology, Danvers, MA, USA) and Fluorescein isothiocyanate- or Alexa 596-conjugated antibody were used as primary and secondary antibodies, respectively. After staining, the cover slips were carefully retrieved with a hooked needle and a fine-tipped tweezer and mounted on the slides using ProLong Gold Antifade Mountant with DAPI (Thermo Fisher Scientific, Waltham, MA, USA). The pictures were taken as described above. For quantification, the coverslip was divided into 4 quadrants, and 3 pictures were captured to represent each quadrant. In each picture, the fluorescence intensity of the positive LC3 signal was quantified by Image J (National Institute of Health, Bethesda, MD, USA) and the number of DAPI-stained nuclei counted using Adobe Photoshop CS5 (Adobe Systems Inc., San Jose, CA, USA). The activity of autophagosome formation was expressed as the fluorescence intensity of LC3 signal divided by the number of cell nuclei.

### Western blots

The cells subjected to Western blot analysis were divided into 2 or 8 groups (n = 6 / group) as described above and cultured in 6-well plates throughout the experiment. Following the corresponding treatments as mentioned above, the cell total proteins were extracted using RIPA Lysis Buffer (Medium, CWBIO, Beijing, China) supplemented with Protease Inhibitor Cocktail (CWBIO, Beijing, China) following the manufacturer’s protocol. Following the total protein concentration measurement as described above, 30 μg of total proteins from each group were resolved in a 12.5% sodium dodecyl sulfate polyacrylamide gel, and transferred to a polyvinylidene difluoride membrane. The blots were washed, blocked with 5% non-fat dry milk or BSA, and incubated with rabbit monoclonal antibodies to LC3A/B (1:1000; Cell Signaling Technology, Danvers, MA, USA) and Beclin 1 (1:1000; Cell Signaling Technology, Danvers, MA, USA) at 4° C overnight. On the next day, the blots were washed and incubated with a goat anti-rabbit HRP-conjugated secondary antibody (1:10000; Abcam, Cambridge, MA, USA) at RT for 2 h. The protein signals were visualized with enhanced chemiluminescence plus reagents (Amersham Biosciences, Piscataway, NJ, USA), and imaged using a Multispectral Imaging System (Biospectrum AC Chemi HR 410, UVP, LLC, Upland, CA, USA). The blots were then stripped and probed with a mouse monoclonal antibody to β-actin (1:5000; Abcam, Cambridge, MA, USA). The optical densities of LC3-I, LC3-II, Beclin 1, and β-actin were quantified by Image J (National Institute of Health, Bethesda, MD, USA). The optical density of Beclin 1 was normalized to that of β-actin. The optical density ratios of LC3-II over LC3-I were calculated.

### Caspase-3/7 activity assay

The cells were divided into 2 or 8 groups (n = 6 / group), cultured in 6-well plates, subjected to the corresponding treatments as abovementioned. The total protein was extracted as described above. Afterwards, caspase-3/7 activity was determined by an Apo-ONE Homogeneous Caspase-3/7 Assay (Promega, Madison, WI, USA) according to the company's protocol. Briefly, 100 μl protein sample or the RIPA Lysis Buffer (serving as empty control; CWBIO, Beijing, China) were incubated with an equal volume of Caspase-3/7 reagent for 16 h at RT in a black 96-well plate (Corning, Corning, NY, USA). The fluorescence intensity was measured by the Infinite 200 PRO Multimode Microplate Reader (excitation 499 nm, emission 521 nm, Tecan Group Ltd., Männedorf, Switzerland). The measured fluorescence intensity was subtracted by that of the empty control, and then normalized to the total protein concentration of the same sample. The fluorescence intensities prior to and after normalization were linearly correlated with the Caspase-3/7 activities according to the company’s protocol.

### Statistics

The statistical analyses were performed using Statistic Program for Social Sciences 20.0 (IBM SPSS Inc., New York, NY, USA). The data were examined by D’Agostino-Pearson omnibus normality test and Levene test to confirm Gaussian distribution and homogeneity of variance, respectively. All data were expressed as Mean ± SEM. The data were compared either by two-tailed Student’s t-test or by One-way ANOVA followed by Tukey post hoc for pairwise comparisons. P value less than 0.05 was considered statistically significant.
